# Neoadjuvant chemoradiotherapy for resectable esophageal cancer: an in-depth study of randomized controlled trials and literature review

**DOI:** 10.7497/j.issn.2095-3941.2014.03.005

**Published:** 2014-09

**Authors:** Xiao-Feng Duan, Peng Tang, Zhen-Tao Yu

**Affiliations:** Department of Esophageal Cancer, Tianjin Medical University Cancer Institute and Hospital, Tianjin 300060, China

**Keywords:** Esophageal cancer (EC), neoadjuvant therapy, chemoradiotherapy, esophagectomy, review, randomized controlled clinical trials

## Abstract

Surgery following neoadjuvant chemoradiotherapy (NCRT) is a common multidisciplinary treatment for resectable esophageal cancer (EC). After analyzing 12 randomized controlled trials (RCTs), we discuss the key issues of surgery in the management of resectable EC. Along with chemoradiotherapy, NCRT is recommended for patients with squamous cell carcinoma (SCC) and adenocarcinoma (AC), and most chemotherapy regimens are based on cisplatin, fluorouracil (FU), or both (CF). However, taxane-based schedules or additional studies, together with newer chemotherapies, are warranted. In nine clinical trials, post-operative complications were similar without significant differences between two treatment groups. In-hospital mortality was significantly different in only 1 out of 10 trials. Half of the randomized trials that compare NCRT with surgery in EC demonstrate an increase in overall survival or disease-free survival. NCRT offers a great opportunity for margin negative resection, decreased disease stage, and improved loco-regional control. However, NCRT does not affect the quality of life when combined with esophagectomy. Future trials should focus on the identification of optimum regimens and selection of patients who are most likely to benefit from specific treatment options.

## Introduction

Esophageal cancer (EC) is the eighth most common cancer and sixth most common cause of death from cancer worldwide^1^. An estimated 482,300 new cases and 406,800 related deaths occurred worldwide in 2008^1^. For decades, surgical resection, the mainstay treatment of EC patients, has had a poor long-term survival rate, even for localized diseases. High local and systemic failure rates prompt us to explore more effective multidisciplinary treatments.

Strong evidence suggests that surgery following neoadjuvant chemoradiotherapy (NCRT) is the most effective combination for locally advanced EC. Furthermore, the results of an updated meta-analysis demonstrate the survival benefit of NCRT over surgery in patients with EC^2^^,^^3^. A recent large randomized trial of NCRT in patients with esophageal or esophagogastric-junction cancer showed a significantly better and disease-free survival without increased post-operative complications and in-hospital mortality^4^. However, not all randomized controlled trials (RCTs) that compare NCRT and surgery have shown encouraging results. Among 11 previous RCTs that investigate the efficiency of NCRT compared with surgery^5^^-^^16^, only 5 have significant survival benefit, including overall survival and/or disease-free survival (**Table 1**), while the other 6 RCTs not having survival advantages (**Table 2**). NCRT for EC is still debated among clinicians with many intractable issues that need to be solved.

**Table 1:** RCTs of NCRT *vs.* surgery alone

Year	Country	*n*	Histology	Radiotherapy (Gy)	Chemotherapy	Sequence	Surgical time (weeks)	Follow-up (months)
1996^8^	Ireland	113	AC	40	CF†	Concurrent	8	10
1997^9^	France	282	SCC	37	C‡	Sequential	2-4	55.2
2002^11^	Ireland	113	AC	40	CF	Concurrent	8	60
2006^14^	Japan	45	SCC	40	CF	Concurrent	5	–
2008^15^	USA	56	AC (75%)	50.4	CF	Concurrent	3-8	72
2009^16^	China	236	SCC	40	CF + mitomycin	Concurrent	2-3	–
2012^4^	Holland	366	AC (75%)	41.4	Carboplatin + paclitaxel	Concurrent	4-6	45.4

†, cisplatin + fluorouracil; ‡, cisplatin; RCTs, randomized controlled trials; NCRT, neoadjuvant chemoradiotherapy; AC, adenocarcinoma; SCC, squamous cell carcinoma.

**Table 2:** RCTs of NCRT *vs.* surgery alone

Year	Country	*n*	Histology	Radiotherapy (Gy)	Chemotherapy	Sequence	Surgical time (weeks)	Follow-up (months)
1992^5^	Norway	78	SCC	35	C† + bleomycin	Sequential	–	–
1994^6^	Thailand	69	SCC	40	CF‡	Concurrent	4	–
1994^7^	France	86	SCC	20	CF	Sequential	6	–
2001^10^	USA	100	AC (75%)	45	CF+ vinblastine	Concurrent	6	98
2004^12^	Korea	101	SCC	45.6	CF	Concurrent	3-4	25
2005^13^	Australia	256	AC (62%)	35	CF	Concurrent	3-6	65

†, cisplatin; ‡, cisplatin + fluorouracil; RCTs, randomized controlled trials; NCRT, neoadjuvant chemoradiotherapy; AC, adenocarcinoma; SCC, squamous cell carcinoma.

In this review, we discuss the following issues through an in-depth study of present literature that compare NCRT with surgery alone: (1) NCRT sample choice; (2) NCRT schemes; (3) NCRT toxic effects and responses; and (4) post-operative complications and long-term survival. We searched PubMed to identify all the RCTs published that directly compares NCRT followed by surgery with surgery alone and excluded abstracts or meeting reports. Finally, 12 RCTs were analyzed in this study.

## Sample choice

EC usually occurs as either squamous cell carcinoma (SCC) in endemic areas or as adenocarcinoma (AC) in non-endemic areas. Sample choice depends on the epidemiological characteristics of EC. Seven studies focusing on SCC were mainly from Asia^12^^,^^14^^,^^16^, France^7^^,^^9^, and Norway^5^. Among them, three trials showed an improved overall survival and/or disease-free survival in patients who received NCRT. Two of these trials were from France^9^ and China^16^ and had the largest sample sizes. Similarly, five studies focusing mainly on AC were from the United States^10^^,^^15^, Australia^13^, Holland^4^, and Ireland^8^^,^^11^. Among them, three trials showed an improved survival in patients who received NCRT. One of these successful trials was from Holland^4^ and had the largest sample size.

In a trial from Australia, subgroup analysis showed that patients with SCC had better progression-free survival than those with non-SCC^13^; however, the histology of SCC was independently associated with shorter survival in another trial^10^. In Dutch trials, the benefit on survival of EC patients with NCRT was consistent across the subgroups according to histologic subtype^4^. A recent meta-analysis^3^ found that NCRT was associated with a significantly improved 1-year (RR=0.86, *P*=0.03), 3-year (RR=0.82, *P*=0.0007), and 5-year (RR=0.83, *P*=0.01) survival time compared with surgery alone. Furthermore, NCRT could improve 3- and 5-year survival outcomes for SCC but not those of AC. The hazard ratio (HR) was 0.78 (*P*<0.0001) for NCRT all-cause mortality, 0.80 (*P*=0.004) for SCC only, and 0.75 (*P*=0.02) for AC only. However, the previous meta-analysis^2^ showed evidence supporting the use of NCRT for both SCC and AC. The difference between the two meta-analyses may have been because of the evidence-based differentiation of RCTs and evaluation criterion. Therefore, NCRT is recommended for both SCC and AC patients. Based on the available evidence, a differentiation of therapy between SCC and AC is not warranted. RCTs with large sample sizes need to focus on a single histological subtype to eliminate the interference caused by tumor heterogeneity.

The esophageal and gastro-esophageal junction AC has something in common. Six RCTs investigated AC, which included esophageal or gastro-esophageal junction AC without strict differentiation. Further studies are still needed to differentiate between the two after NCRT. Siewert^17^^,^^18^ classified the gastroesophageal junction AC according to their location in tumors of the distal esophagus (AEG type I), tumors of the cardia or gastro-esophageal junction (AEG type II), and sub-cardial gastric carcinoma (AEG type III). Recommendations based on the Siewert classification of the gastroesophageal junction AC were as follows: surgery following NCRT for operable AEG type I or II tumors and gastrectomy following perioperative gastric cancer chemotherapy in AEG type III tumors that are localized in the stomach. Even if complete remission occurred during pre-operative therapy, surgery will be performed as planned^19^.

## NCRT schemes

Most chemotherapy regimens are based on cisplatin, fluorouracil (FU), or both (CF) (**Table 3**). The combination of FU and cisplatin has been a standard radio-sensitizing regimen for several decades with an efficiency of about 25% to 35%. Paclitaxel is a promising agent against EC. A single activity reaches 32% when administrated alone^20^. Paclitaxel has been widely used in concurrent NCRT in recent years^21^^-^^23^. Paclitaxel and cisplatin regimens have achieved better efficiency of about 50% to 60% in a neoadjuvant and definitive setting for advanced EC^24^. A phase II study of concurrent CRT with paclitaxel and cisplatin for inoperable esophageal SCC observed better survival rates, with 1-, 2-, 3-, and 4-year survival rates of 75%, 54%, 41%, and 29%, respectively^25^.

**Table 3:** NCRT schemes

Year	Radiotherapy	Chemotherapy
1992^5^	35 Gy, 1.75 Gy fraction over 4 weeks	Two cycles: cisplatin 20 mg/m^2^ days 1-5; bleomycin 5 mg/m^2^ days 1-5
1994^7^	20 Gy, 2 Gy fraction over 12 days	Two cycles: cisplatin 100 mg/m^2^ day 1; fluorouracil 600 mg/m^2^ days 2-5 and 22-25
1994^6^	40 Gy, 2 Gy per fraction over 4 weeks	Two cycles: cisplatin 100 mg/m^2^ day 1; fluorouracil 1,000 mg/m^2^ days 1-4
1996^8^	40 Gy in 15 fractions over 3 weeks	Two cycles: cisplatin 75 mg/m^2^ day 7; fluorouracil 15 mg/kg days 1-5
1997^9^	37 Gy, 3.7 Gy fraction over 2 weeks	Two cycles: cisplatin 80 mg/m^2^ days 0-2
2001^10^	45 Gy, 1.5 Gy fraction over 3 weeks	Two cycles: cisplatin 20 mg/m^2^ days 1-5; fluorouracil 300 mg/m^2^ days 1-21; vinblastine 1 mg/m^2^ days 1-4
2002^11^	40 Gy in 15 fractions over 3 weeks	Two cycles: cisplatin 75 mg/m^2^ day 7; fluorouracil 15 mg/kg days 1-5
2004^12^	45.6 Gy, 1.2 Gy per fraction over 4 weeks	Two cycles: cisplatin 60 mg/m^2^ day 1; fluorouracil 1,000 mg/m^2^ days 3-5
2005^13^	35 Gy in 15 fractions over 3 weeks	One cycle: cisplatin 80 mg/m^2^ day 1; fluorouracil 800 mg/m^2^ days 2-5
2006^14^	40 Gy, 2 Gy fraction over 4 weeks	One cycle: cisplatin (7 mg over 2 h); 5-fluorouracil (350 mg over 24 h)
2008^15^	50.4 Gy, 1.8 Gy per fraction over 5.6 weeks	Two cycles: cisplatin 60 mg/m^2^ day 1; fluorouracil 1,000 mg/m^2^ days 3-5
2009^16^	40 Gy, 2 Gy per fraction over 4 weeks	One cycle: cisplatin 20 mg/m^2^ days 1-5; 5-fluorouracil 500 mg/m^2^ days 1-5 ; mitomycin 10 mg/m^2^ day 1
2012^4^	41.4 Gy in 23 fractions, 5 days per week	Weekly administration for 5 weeks: carboplatin (achieve an area under the curve of 2 mg per milliliter per minute); paclitaxel (50 mg per square meter of body surface area)

NCRT, neoadjuvant chemoradiotherapy.

NCRT, utilizing concurrent paclitaxel and radiotherapy followed by surgery, resulted in a significant pathologic complete response (38%) or minimal residual disease (31%)^26^. Adverse effects were generally tolerated. A comparison of two NCRT regimens in patients with potentially curable EC proved that the carboplatin/paclitaxel/41.4 Gy regimen caused less toxicity compared with the cisplatin/5-FU/50.4 Gy regimen, with an insignificant difference in response rates and long-term survival^27^. In the Dutch trial^4^, patients who had NCRT with paclitaxel and carboplatin weekly for 5 weeks with 41.4 Gy radiotherapy experienced a survival benefit unlike patients who had surgery alone. Another NCRT study^28^ used paclitaxel (135 mg/m^2^ on day 1) and cisplatin (20 mg/m^2^ on days 1-3). Even with a different paclitaxel schedule, a survival benefit was reported among patients who had NCRT, unlike patients who had surgery alone. Other clinical trials also provided evidence that a chemotherapy regimen containing paclitaxel rather than 5-FU was well tolerated. The survival data also favored paclitaxel against other previously reported combinations^29^^-^^31^. Therefore, further development of taxane-based CRT schedules and additional studies in new chemotherapy combinations are warranted.

Significant gain in long-term survival improvement after adding radiotherapy to pre-operative chemotherapy is still debated because of the limited data comparing pre-operative CRT and pre-operative chemotherapy for EC. Stahl *et al*.^32^ randomly assigned 126 patients to NCRT and chemotherapy groups to evaluate the value of adding radiotherapy in pre-operative chemotherapy with a median observation time of 46 months; a total of 119 patients were eligible and evaluated. The number of patients who underwent complete tumor resection was similar between treatment groups (69.5% *vs*. 71.5%). Patients in the NCRT group had a significantly higher probability of showing a pathologic complete response (15.6% *vs*. 2.0%) or tumor-free lymph nodes (64.4% *vs*. 37.7%) at resection. Pre-operative radiotherapy improved the 3-year survival rate from 27.7% to 47.4% (*P*=0.07). Post-operative mortality did not significantly increase in the NCRT group (10.2% *vs*. 3.8%). A short duration and lack of statistical significance limited the study, but results pointed to a survival advantage for NCRT compared with pre-operative chemotherapy in AC of esophagogastric junction. Swisher *et al*.^33^ also reported that in sequential phase II/III trials involving locoregionally advanced EC patients, NCRT was associated with improved overall and disease-free survival rates (*P*=0.046 and *P*=0.015, respectively) and increased pathological complete response *P*<0.001 compared with pre-operative chemotherapy. However, a recent phase II clinical trial^34^ showed NCRT with regimens of cisplatin, and 5-FU did not show an improved survival benefit compared with pre-operative chemotherapy, which had the same drugs in patients with resectable AC of the esophagus and gastroesophageal junction. The histopathological response rate (NCRT 31% *vs*. chemotherapy 8%, *P*=0.01) and R1 resection rate (CRT 0% *vs*. chemotherapy 11%, *P*=0.04) favored those of NCRT recipients.

The value of adding surgery to CRT in patients with locally advanced EC has been evaluated in clinical trials^35^^-^^38^. The FFCD 9102 trial concluded that the addition of surgery after NCRT had no benefit in patients with locally advanced ECs, especially SCC, which responded to chemoradiation, compared with patients continuing additional CRT^37^. Furthermore, another clinical trial demonstrated a significantly increased treatment-related mortality in surgery groups (from 12.8% to 3.5% in the CRT group) and had no significant long-term outcomes with a median follow-up of 10 years^39^. Stahl *et al*.^36^ evaluated 172 patients with locally advanced esophageal SCC. The patients received induction chemotherapy followed by CRT and were randomized into groups followed with and without surgical intervention. The patients who had surgery had a better 2-year progression-free survival of 64.3% than the CRT group with 40.7%. Adding surgery to chemoradiotherapy improved local tumor control but did not increase survival of patients with locally advanced esophageal SCC.

Previously, 132 consecutive patients with clinical stage II or III EC treated with concurrent CRT were reviewed retrospectively. Patients treated with NCRT and esophagectomy had statistically significant superior 5-year loco-regional control (67.1% *vs*. 22.1%), disease-free survival (40.7% *vs*. 9.9%), and 5-year overall survival (52.6% *vs*. 6.5%) rates and median survival time (62 *vs*. 12 months) compared with patients treated with CRT alone^35^. A recent study^38^ evaluated the clinical results of 100 patients with T_4_ SCC of the esophagus after either a definitive CRT or esophagectomy following down-staging by pre-operative CRT. The 5-year survival rates were 19% and 42% in definitive CRT groups and surgery group, respectively. A recent study^40^ also confirmed that long-term survival could be expected after multimodal therapy, and esophagectomy was therefore a valid treatment option when down-staging was achieved.

## NCRT toxic effects and responses

The most common toxic effects during NCRT are fatigue^4^, nausea, vomiting^5^, esophagitis^13^, and hematologic toxicity^10^^,^^15^. **Table 4** shows that NCRT decreased the number of patients undergoing surgery. In the Dutch trial, 168 patients (94%) underwent surgery in the NCRT group, whereas 186 (99%) underwent surgery in the surgery group (*P*=0.01). The main reasons for not having surgery were disease progression during treatment and the decision of the patients^4^. In another randomized clinical trial, 48 patients (96%) underwent surgery in the surgery group, whereas 35 of 51 patients (69%) in the NCRT group underwent surgery (*P*<0.01). The patients who did not have surgery refused treatment^12^. The refusal may be because of the good responses to CRT and the potential for a high level of associated morbidity. Other patients were unable to undergo surgery because of disease progression.

**Table 4:** Surgical characteristics of NCRT group *vs*. surgery alone group

Year	Toxic effects	Surgery (%, *P*)	R0 resection (%, *P*)	pCR† (%)	Positive nodes (%, *P*)	Illustration
1992^5^	Nausea, vomiting, leucopenia, thrombocytopenia	72/93, –	55/37, 0.079	–	–	–
1994^7^	–	85/93, –	–	–	–	–
1994^6^	–	74/100, –	–	27	–	–
1996^8^	–	83/80, –	–	25	–	–
1997^9^	–	97/99, –	81/69, 0.017	26	37/55, 0.03	Low T, N stage
2001^10^	Leucopenia, malnutrition, neutropenic fever	94/100, –	84/88, –	28	–	Low local failure
2004^12^	–	69/96, 0.01	100/87.5, 0.037	43	37/78, <0.001	Low T, N, TNM stage
2005^13^	Esophagitis, nausea, vomiting, infections	82/85, –	80/59, 0.0002	16 (AC 9/SCC 27, *P*=0.02)	43/67, 0.003	Lowly, v invasion‡
2006^14^	–	90/100, –	–	–	55/74, >0.05	–
2008^15^	Hematologic toxicity, esophagitis, infection	–	–	–	–	–
2009^16^	–	–	98.3/77.3, <0.001	22.3	–	–
2012^4^	Fatigue, leucopenia, thrombocytopenia	94/99, 0.01	92/69, <0.001	29 (AC 23/SCC 49, *P*=0.008)	31/75, <0.001	–

†, pathological complete response; ‡, lower lymphatic and venous invasion; pCR, pathological complete response rate; NCRT, neoadjuvant chemoradiotherapy; AC, adenocarcinoma; SCC, squamous cell carcinoma.

**Table 4** shows that NCRT increased the number of patients who underwent R0 resection. Six out of seven trials, with available data, showed significant differences. In the Dutch trial^4^, an R0 resection was achieved in 148 out of 161 patients (92%) in the NCRT group, whereas R0 resection was achieved in 111 out of 161 (69%) patients in the surgery group (*P*<0.001). Another clinical trial demonstrated that all patients who underwent esophagectomy in the NCRT group achieved R0 resection, which was curative in more patients (*P*=0.037)^12^.

Resected specimens were pathologically assessed. The pathological complete response rate (pCR) ranged from 16% to 43%, with a median of 26.5%. A pCR of 23% was observed in 28 out of 121 patients with AC and 49% in 18 out of 37 patients with SCC (*P*=0.008) in the Dutch trial4; 9% with AC *vs*. 27% with SCC in another trial (*P*=0.02)^13^. Therefore, patients with esophageal SCC have good responses to CRT. In addition, the number of patients with positive lymph nodes decreased in five clinical trials with available data. Metastasis was found in the lymph nodes of resected specimen in 50 patients (31%) of the NCRT group, whereas metastasis was observed in 120 patients (75%) in the surgery group (*P*<0.001)^4^. Two other studies pathologically revealed a significantly lower stage of disease in T, N, and combined TNM stages^9^^,^^12^. The frequency of lymphatic and venous invasion^14^ and local failure rate^10^ was also significantly lower in the NCRT group. NCRT offers a great opportunity for margin negative resection, improved loco-regional control, and decreased disease stage.

We determined the population group who are most likely to benefit from NCRT. Histology and TNM stage^29^, pCR^41^^-^^43^, and R0 resection^44^ are identified as independent prognostic indicators for EC patients who underwent NCRT. The benefit is highly dependent on the tumor response to NCRT^45^^-^^47^. Recurrence developed in 24 out of 62 patients (38.7%) with pCR and 70 out of 126 patients (55.6%) without pCR (*P*=0.044)^48^. Locoregional recurrence (LRR) with or without synchronous distant metastases occurred in 8 patients (13%) in the pCR group and in 31 patients (24.6%) in the non-pCR group (*P*=0.095)^48^. The overall 5-year survival rate was significantly higher in the pCR group than in the non-pCR group (52% *vs*. 33.9% respectively; *P*=0.019)^48^. Although pCR is favorable for survival, the method is not a cure or a complete locoregional disease control.

A recent study^49^ identified pre-therapeutic hemoglobin (Hb) level as an independent and useful marker for predicting pathologic tumor responses. Only 17.1% of patients with Hb levels ≤13 g/dL responded to treatment, whereas 48.8% of patients with a level of >13 (*P*=0.0002) responded. The patients had a 5-year overall survival rates of 40.9% and 58.9%, respectively (*P*=0.048). Other studies confirmed that the Hb level was associated with sensitivity^50^, loco-regional control^51^^,^^52^, and survival^53^^,^^54^ after radiation therapy for EC, regardless of chemotherapy. Thus, Hb levels can serve as a useful marker for tailoring the optimal therapies of individual patients with advanced EC.

Some studies have attempted to predict the effects of NCRT on the basis of assessments of biopsy samples including protein and gene expression^55^^-^^57^. Musashi-1, a stem cell marker, was used to stain biopsy and surgically resected tissue specimens to examine the relationship of the staining intensity with response to NCRT, recurrence, and prognosis. The results suggested the possibility of Musashi-1 as a candidate marker for the histological response and prognosis of EC^58^. Analysis of selected microRNA (miRNA) of pre-therapeutic and post-therapeutic biopsies characterized miRNA profiles of responders and non-responders in the NCRT therapy of locally advanced EC. MiR-192 and miR-194 in pre-therapeutic biopsies were considered indicators of major histopathologic regressions^59^. However, findings could not clinically distinguish poor responders well.

Esophagectomy was traditionally recommended to perform within 8 weeks after NCRT. A recent study retrospectively studied the effect of delayed surgery in 276 EC patients treated with NCRT and concluded that the method might be hazardous, especially in patients demonstrating good responses^60^. The amount of residual cancer increased significantly after a longer surgical interval (*P*=0.024). Survival also decreased after a longer surgical interval (5-year overall survival: 50% *vs*. 35%; *P*=0.038). Esophagectomy should be performed after NCRT within 8 weeks, especially in patients with good responses.

## Post-operative complications and long-term survival

Neoadjuvant therapies are associated with toxicity, which can contribute to subsequent post-operative morbidity and mortality. Conflicting evidence exists regarding the effect of these neoadjuvant approaches on NCRT outcomes compared with the outcomes in patients treated by surgery alone. Some investigators^61^^,^^62^ have reported a higher post-operative mortality after NCRT with surgery compared with surgery alone. In 9 out of 10 clinical trials with available data, post-operative complications were similar without significant differences in the two treatment groups, and in-hospital mortality was significantly different in only one trial (**Table 5**). Little association between risk of post-operative morbidity and mortality and neoadjuvant interventions was found. A meta-analysis based on 23 relevant studies showed that no increase in morbidity or mortality was attributable to neoadjuvant therapy. Subgroup analysis of NCRT for SCC suggested an increased risk of total post-operative mortality and treatment-related mortality compared with surgery alone. Care should be taken with NCRT in esophageal SCC, where an increased risk of post-operative mortality and treatment-related mortality was apparent^63^. A study^64^ compared the surgical outcomes between 114 patients who did not receive neoadjuvant therapy (group 1) and 92 others who received NCRT (group 2). The pre-operative and surgical factors that influenced post-operative morbidity were assessed to determine the effect of NCRT on morbidity and mortality after esophagectomy via cervical, right transthoracic, and abdominal approaches. The overall post-operative morbidity rates were 44.7% and 55.4% in groups 1 and 2, respectively (*P*=0.13). The rates of anastomotic leak (8.8% *vs*. 16.3%; *P*=0.10), pneumonia (9.6% *vs*. 13.0%; *P*=0.44), recurrent nerve palsy (15.8% *vs*. 10.9%; *P*=0.31), and all other complications did not significantly differ between the groups. Multivariable analysis revealed cervical lymph node dissection as the sole independent covariate for overall morbidity. Furthermore, a history of cardiovascular disease, retrosternal reconstruction route, and a longer surgical duration were independent covariates for anastomotic leakage. Old age and a lower body mass index were independent covariates for pneumonia. However, whether or not patients received NCRT was irrelevant. A study^65^ also confirmed NCRT followed by esophagectomy in elderly patients as a safe treatment modality.

**Table 5:** Post-operative complications and long-term survival of NCRT group *vs*. surgery alone group

Year	Morbidity	Mortality	Median survival (months)	Overall survival (1; 2; 3; 4; 5 years) (NCRT/S, %)	Sig. (*P*)	DFS†	Prognostic factors
1992^5^	NS‡	NS	–	34/39; 13/23; 9/17; –; –	NS	–	–
1994^7^	NS	NS	–	46.6/46.7; –; 19.2/13.8; –; –	NS	NS	–
1994^6^	NS	NS	9.7/7.4	49/39; –; –; –; 24/10	NS	–	pCR
1996^8^	–	–	32/11	52/44; 37/26; 32/6; –; –	0.001	–	–
1997^9^	NS	0.012	–	–	NS	0.003	Location, R0, N stage
2001^10^	–	–	17.6/16.9	72/58; 30/16; –; –	NS	NS	pCR, size, age, histology
2002^11^	–	–	34/14	–	0.0001	–	–
2004^12^	NS	NS	27.3/28.2	–; 57/55; –; –; –	NS	NS	Weight loss
2005^13^	NS	NS	22.2/19.3	–	NS	NS	Histology, grade, age
2006^14^	NS	NS	–	–; –; –; –; 57/41	NS	0.022	Tumor grade
2008^15^	NS	NS	4.48/1.79 years	–; –; –; –; 39/16	0.002	0.007	–
2009^16^	–	NS	–	–; –; 73.73/53.38; –; –	<0.05	–	–
2012^4^	NS	NS	49.4/24.0	82/70; 67/50; 58/44; –; 47/34	0.003	<0.001	–

†, disease-free survival; ‡, not significant; NCRT, neoadjuvant chemoradiotherapy; pCR, pathological complete response rate.

A total of 5 out of 12 trials showed a significant overall survival benefit, and disease-free survival benefit was found in 4 out of 8 trials. **Table 5** shows a superior overall survival in both groups, which is close to previously reported randomized trials. The survival of patients treated with surgery alone was improved, owing to the ongoing improvements in surgical techniques, patient selection, and staging methods over the years. Therefore, the differences in long-term survival in the recent four trials between 2006 and 2012 were not because of the poor survival in the surgery group but could clearly be attributed to the improved survival in the NCRT group. The results of the updated meta-analysis provided strong evidence for the survival benefit of NCRT over surgery alone in patients with EC^2^^,^^3^. Twelve RCTs were randomized comparisons of NCRT versus surgery alone (*n*=1,854) in patients with resectable EC. The HR for all-cause mortality in NCRT was 0.78 (95% CI, 0.70-0.88; *P*<0.0001); 0.80 for SCC only (95% CI, 0.68-0.93; *P*=0.004); and 0.75 for AC (95% CI, 0.59-0.95; *P*=0.02).

A recent study^66^ analyzed the recurrence patterns in patients with cancer of esophagus or gastroesophageal junction and treated with NCRT and surgery or surgery alone. After a minimum follow-up of 24 months (median, 45 months), the overall recurrence rate in the surgery group was 58% versus 35% in the CRT plus surgery group. NCRT reduced LRR from 34% to 14% (*P*<0.001) and peritoneal carcinomatosis from 14% to 4% (*P*<0.001). A small but significant effect on hematogenous dissemination in favor of the CRT group (35% *vs*. 29%; *P*=0.025) was found. LRR occurred by 5% within the target volume, by 2% in the margins, and by 6% outside the radiation target volume. In 1%, the exact site in relation to the target volume was unclear. Only 1% had an isolated in-field recurrence after CRT plus surgery. Hence, NCRT in patients with EC reduced LRR and peritoneal carcinomatosis. Recurrence within the radiation target volume occurred by only 5% and is mostly combined with out-field failures.

Previous studies demonstrated a negative influence of esophagectomy on health-related quality of life (HQoL)^67^^-^^69^. Yamashita *et al*.^70^ studied the effect of chemoradiotherapy treatment on patients’ HQoL and late toxicities. They concluded that the HQoL score deteriorates before treatment because of acute chemoradiotherapy-related complications, but recovers in four to five months. The FFCD 9102 trial, a randomized multicenter phase III trial^71^, compared the longitudinal HQoL between chemoradiation with or without surgery in patients with locally advanced resectable esophageal SCC. HQoL scores at the first follow-up were worse in patients with surgery, whereas the longitudinal HQoL study showed no difference between treatments. Furthermore, the longitudinal HQoL was not different among survivors after 2 years of follow-up. Patients who responded to induction chemoradiation, surgery, and continuation of chemoradiation received the same effect on HQoL as in patients who had locally advanced resectable EC. A recent study^72^ examined HQoL during pre-operative chemotherapy/chemoradiotherapy treatments and compared the post-operative recovery of HQoL in patients undergoing combined treatments with surgery alone. Deterioration in most aspects of HQoL occurred during pre-operative chemotherapy. Patients who proceeded to concomitant radiotherapy further deteriorated with specific problems of reflux symptoms and role functions (*P*<0.01). After neoadjuvant treatment but before surgery, HQoL returned to baseline levels. Six weeks after surgery, patients reported marked reductions in physical, role, and social function (*P*<0.01) and an increase in fatigue, nausea, emesis, pain, dyspnea, appetite loss, and coughing (*P*<0.01). Pre-operative treatment did not hamper the recovery of HQoL, and patients who had undergone neoadjuvant treatment reported fewer problems with post-operative nausea, emesis, and dysphagia, unlike those who had undergone surgery alone. Therefore, pre-operative chemotherapy or chemoradiotherapy had a negative effect on HQoL, which was restored in patients proceeding to surgery. Neoadjuvant treatment did not impair the recovery of HQoL after esophagectomy. These results supported the use of neoadjuvant treatment before surgery.

## Conclusion

Pre-operative chemoradiotherapy followed by surgery is the most common approach for resectable EC, even though this approach has been debated for several decades. However, NCRT offers an undeniable opportunity for clinical down-staging, margin negative resection, improved loco-regional control, and increased survival and should be an optional treatment paradigm. The majority of the available evidence currently reveals that only selected locally advanced EC is likely to benefit from neoadjuvant therapy. Future trials should focus on the identification of the optimum regimen and should attempt to identify and select the patients most likely to benefit from specific treatment options.
